# Distal tibial oblique osteotomy for reconstruction of ankle joint congruity and stability

**DOI:** 10.1016/j.jcot.2021.101588

**Published:** 2021-09-04

**Authors:** Shota Harada, Tsukasa Teramoto, Nobuyuki Takenaka, Takashi Matsushita

**Affiliations:** a Department of Traumatology, Fukushima Medical University, Trauma & Reconstruction Center, Southern TOHOKU General Hospital, Japan; b N-ASAMI (Nagasaki-Association for the Study and Application of the Methods for Ilizarov), Japan

**Keywords:** Distal tibial oblique osteotomy, Ankle osteoarthritis, Dynamic stability, Joint congruity, Supramalleolar osteotomy, Arthroplasty

## Abstract

Teramoto distal tibial oblique osteotomy (DTOO) is a joint-preserving surgery for ankle osteoarthritis (AOA). However, there are few articles on the radiological assessment of DTOO. The purpose of this study was to report the clinical outcomes and radiological evaluations of weight-bearing radiographs before and after DTOO.

We retrospectively reviewed 52 patients who underwent DTOO between 2007 and 2018. We recorded the Tanaka–Takakura classification, fixation methods, Japanese Society for Surgery of the Foot Ankle/Hindfoot Scale (JSSF scale), and complications. The tibial articular surface angle (TAS), medial malleolar angle (MMA), tibial lateral surface angle (TLS), talar tilt angle (TTA), and tibiotalar surface angle (TTS) were evaluated using weight-bearing ankle radiographs.

The median patient age was 66 years, and the mean follow-up duration was 46 ± 23 months. Two stage 2, 9 stage 3a, 30 stage 3b, and 11 stage 4 according to the Tanaka–Takakura classification were performed using DTOO. The JSSF scale improved significantly from 39.9 ± 13.8 before surgery to 87.2 ± 7.5 after surgery. Seven cases were fixed using a locking plate, and 45 cases were fixed using a circular external fixator. The TAS, MMA, TLS, TTA, and TTS significantly changed before and after DTOO.

Radiological evaluation indicated that DTOO influences talar behavior during weight-bearing, and improves the clinical outcomes of AOA.

## 1 Introduction

Degenerative ankle osteoarthritis (AOA) is relatively rare compared with that in the hip and knee. However, Japanese people suffer AOA more frequently compared with Western countries because of their individual cultures, lifestyles, and congenital tibia vara,^1^ so we Japanese orthopedic surgeons tend to experience AOA more often than Western surgeons. The treatment of AOA at the progressive or end stage is controversial. In general, ankle arthrodesis (AA) or total ankle replacements (TAR) are indicated for these patients, but orthopedic surgeons tend to hesitate to perform AA due to the residual range of ankle motion or adjacent arthritis^2^^,^^3^ and are also concerned with the relatively poor prognosis of TAR compared with total hip replacement or total knee replacement.^4^ Prof. Teramoto devised distal tibial oblique osteotomy (DTOO) as an ankle joint-preserving surgery for AOA in 1994^5^ and first reported this procedure in English in 1996,^6^ and subsequently studies have been published since.^5^^,^^7^^,^^8^ We and our colleagues have performed DTOO for progressive or end-stage AOA.

This procedure is completely different from low tibial osteotomy (LTO) at the point of obtaining joint congruity and stability. The purpose of this study was to report the concepts, operative techniques, clinical outcomes, and radiological evaluations of DTOO.

## 2 Patients and methods

### 2.1 Patients

We retrospectively reviewed data between 2007 and 2018. The inclusion criteria were as follows: (1) stage 2, 3a, 3b, and 4 adult AOA according to the Tanaka–Takakura classification^9^^,^^10^ with ankle pain, (2) a follow-up of less than 2 years, and (3) a range of ankle motion less than 10°. Patients with ankle infection, gout, rheumatoid arthritis, neoplastic arthropathy, Charcot arthropathy, neuromuscular dysfunction, and absent weight-bearing radiographs before and after surgery were excluded.

**Fig. 1:**
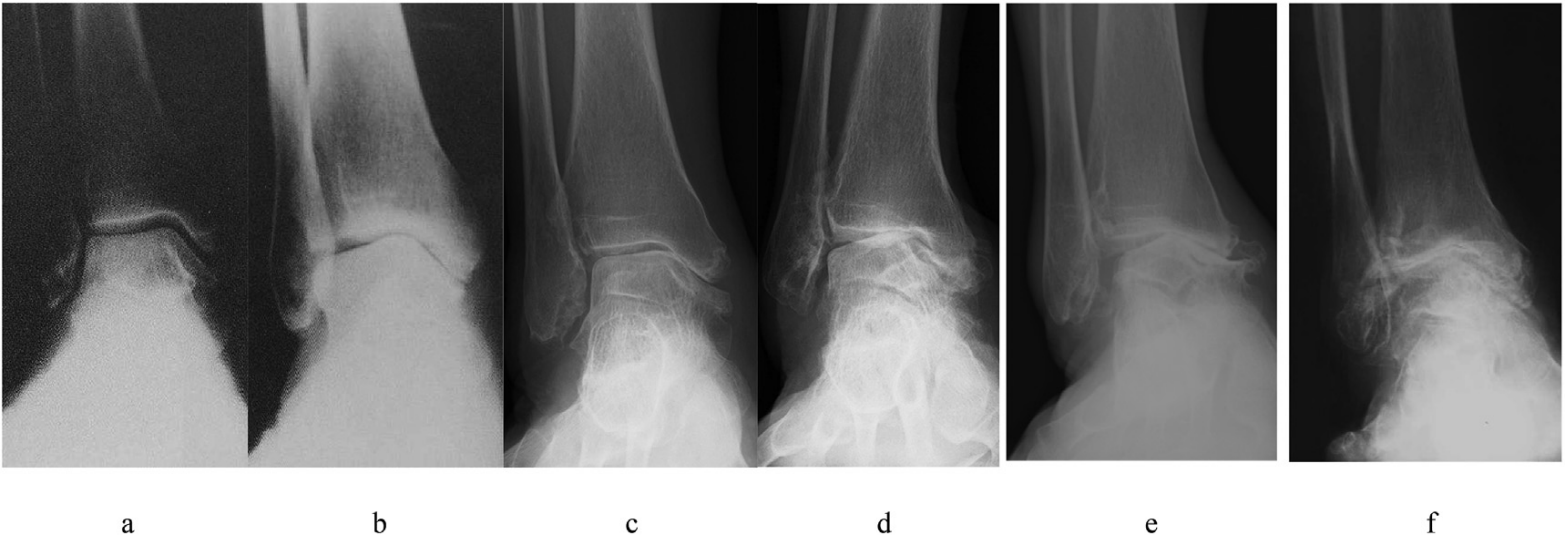
Tanaka-Takakura classification for ankle osteoarthritis with varus deformity. Although both Fig. 1d and e are same stage 3b according to Tanaka-Takakura classification, these are discriminable regarding medial talar shift. **a**. Stage 1: Early sclerosis or osteophytes formation without narrowing of ankle joint. **b**. Stage 2: Narrowing of the ankle joint space medially. **c**. Stage 3a: Disappearance of the ankle joint space limited to the medial malleolus. **d**. Stage 3b: Disappearance of the ankle joint space extended to the roof of the dome of the talus (without medial talar shift). **e**. Stage 3b: Disappearance of the ankle joint space extended to the roof of the dome of the talus (with medial talar shift). **f**. Stage 4: Disappearance of whole ankle joint space.

A total of 52 patients (11 men and 41 women) who underwent DTOO with a median age of 66 years and mean follow-up duration of 46 ± 23 months were included. There were two patients in stage 2, 9 patients in stage 3a, 30 patients in stage 3b, and 11 patients in stage 4 according to the Tanaka–Takakura classification (Fig. 1). Seven patients were fixed using a plate, and 45 patients were fixed using a circular external fixator.

### 2.2 Clinical and radiological analysis

Clinical outcomes were assessed using the Japanese Society for Surgery of the Foot Ankle/Hindfoot Scale (JSSF scale),^11^^,^^12^ and complications necessary for surgical procedures (excluding implant removal) were recorded.

**Fig. 2:**
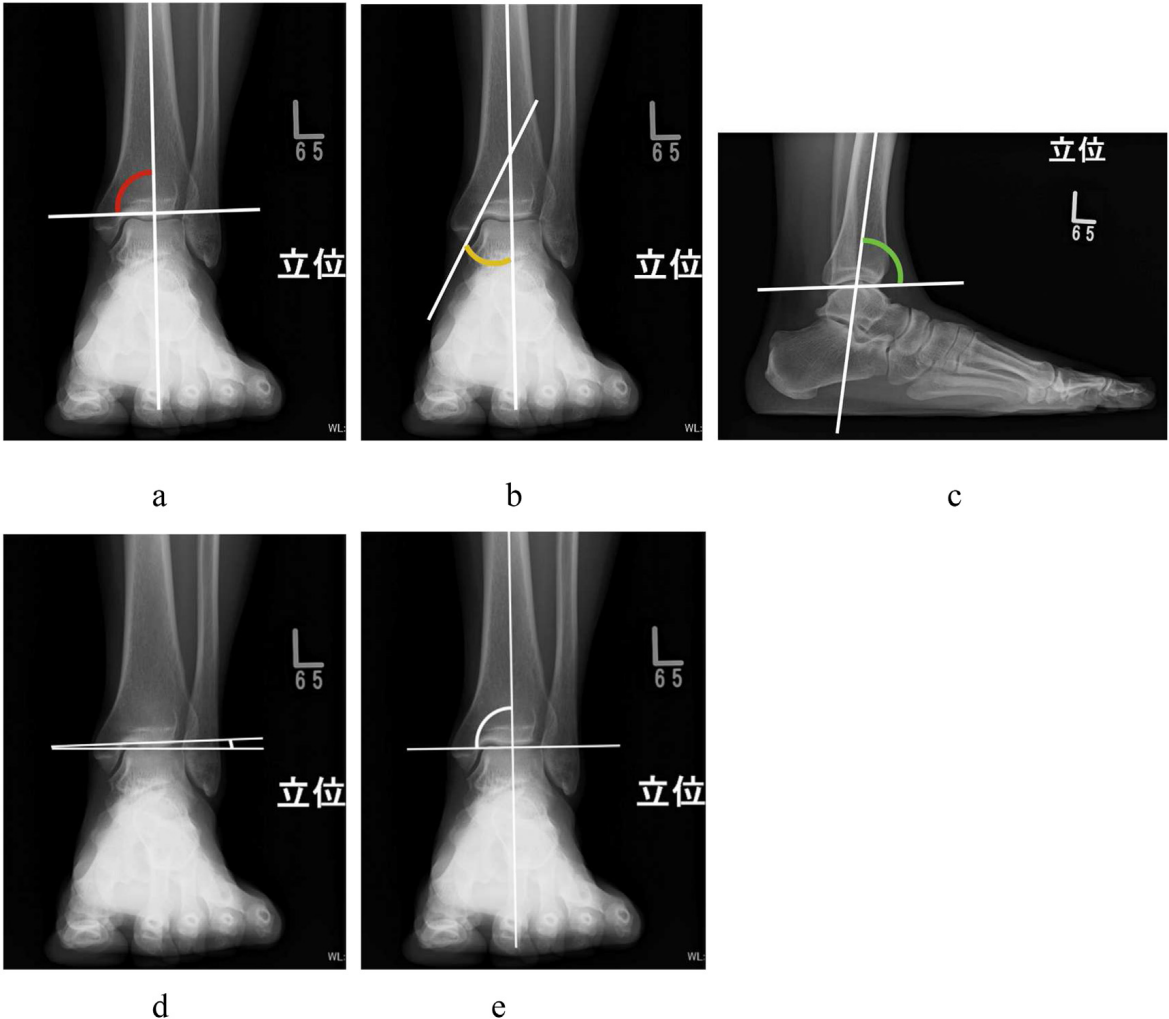
Radiological evaluation parameter on weight-bearing radiograph. **a**. Tibial articular surface angle (TAS): the angle between the axis of the distal one-third part of the tibia and the line along the articular surface of the tibial plafond (mean ± SD = 87.7 ± 3.0°^13^). **b**. Medial malleolar angle (MMA): the angle between the axis of the distal one-third part of the tibia and the line along the articular surface of the medial malleolus (mean ± SD = 23.0 ± 4.7°^13^). **c**. Tibial lateral surface angle (TLS): the angle between the axis of the distal one-third part of the tibia and the line joining the anterior and posterior edges of the tibial plafond(mean ± SD = 81.1 ± 2.2°^13^).**d**. Talar tilt angle (TTA): the angle between the line along the articular surface of the tibial plafond and the line along the superior surface of the trochlea of the talus (normally less than 4°^16^). **e**. Tibiotalar surface angle (TTS): the angle between the axis of the distal one-third part of the tibia and the line along the superior surface of the trochlea of the talus (normally about 90°).

The tibial articular surface angle (TAS),^10^^,^^13^ medial malleolar angle (MMA),^13^ tibial lateral surface angle (TLS),^10^^,^^13^ talar tilt angle (TTA), and tibiotalar surface angle (TTS) were measured on weight-bearing ankle radiographs before and after DTOO. Fig. 2 shows each parameter of the weight-bearing ankle radiograph. A CT scan was done routinely to detect osteophytes and to evaluate three-dimensional figuration. Although we have not taken MRI routinely in past days, MRI has a possibility to be an effective modality to look for signal changes in tibial plafond and talus before and after DTOO.

### **2.3** Surgical technique

The patient was placed in the supine position under general anesthesia. An epidural anesthesia/nerve block was applied as necessary.

#### 2.3.1 Fluoroscopy-assisted stress testing of the ankle joint

**Fig. 3:**
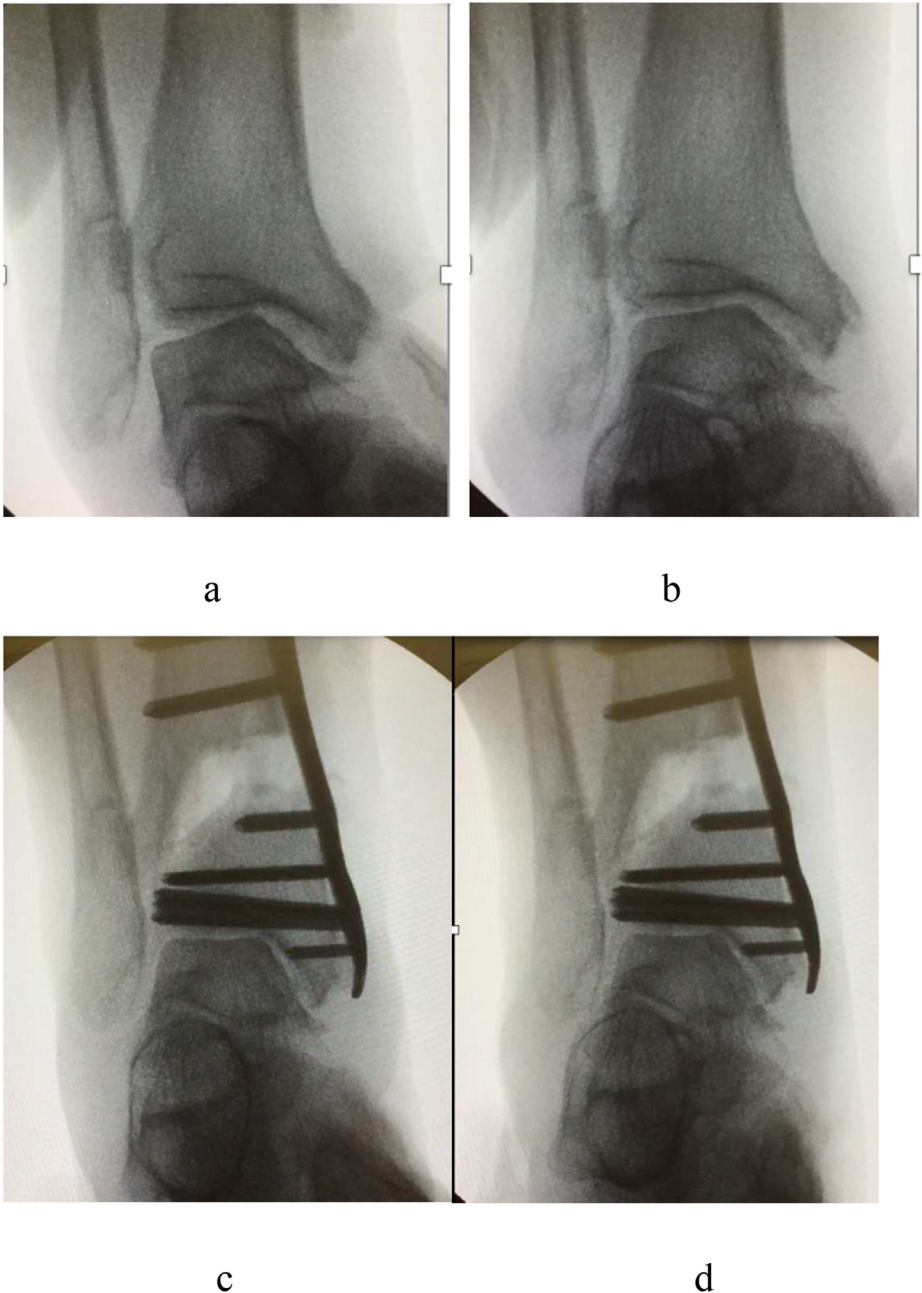
Ankle joint varus–valgus stress test under fluoroscopy (AP views). **a**. Varus stress (before surgery). **b**. Valgus stress (before surgery). Varus–valgus dynamic instability exists before surgery. **c**. Varus stress (after surgery). **d**. Valgus stress (after surgery). Varus–valgus dynamic instability disappears after distal tibial oblique osteotomy.

The dynamic instability of the ankle joint was examined under fluoroscopy before surgery. Specifically, in anterior-posterior (AP) views, the dynamic instability was evaluated using the following stress tests: varus–valgus, internal–external rotation, and dorsiflexion–plantar flexion stress tests. In lateral views, dynamic instability was evaluated with the anterior–posterior drawer test and dorsiflexion–plantar flexion stress test. Data regarding preoperative dynamic instability are essential for determining the extent to which ankle stability is sufficiently restored after widening the osteotomy gap. Fig. 3 shows the stress test results before and after DTOO.

#### 2.3.2 Osteotomy

**Fig. 4:**
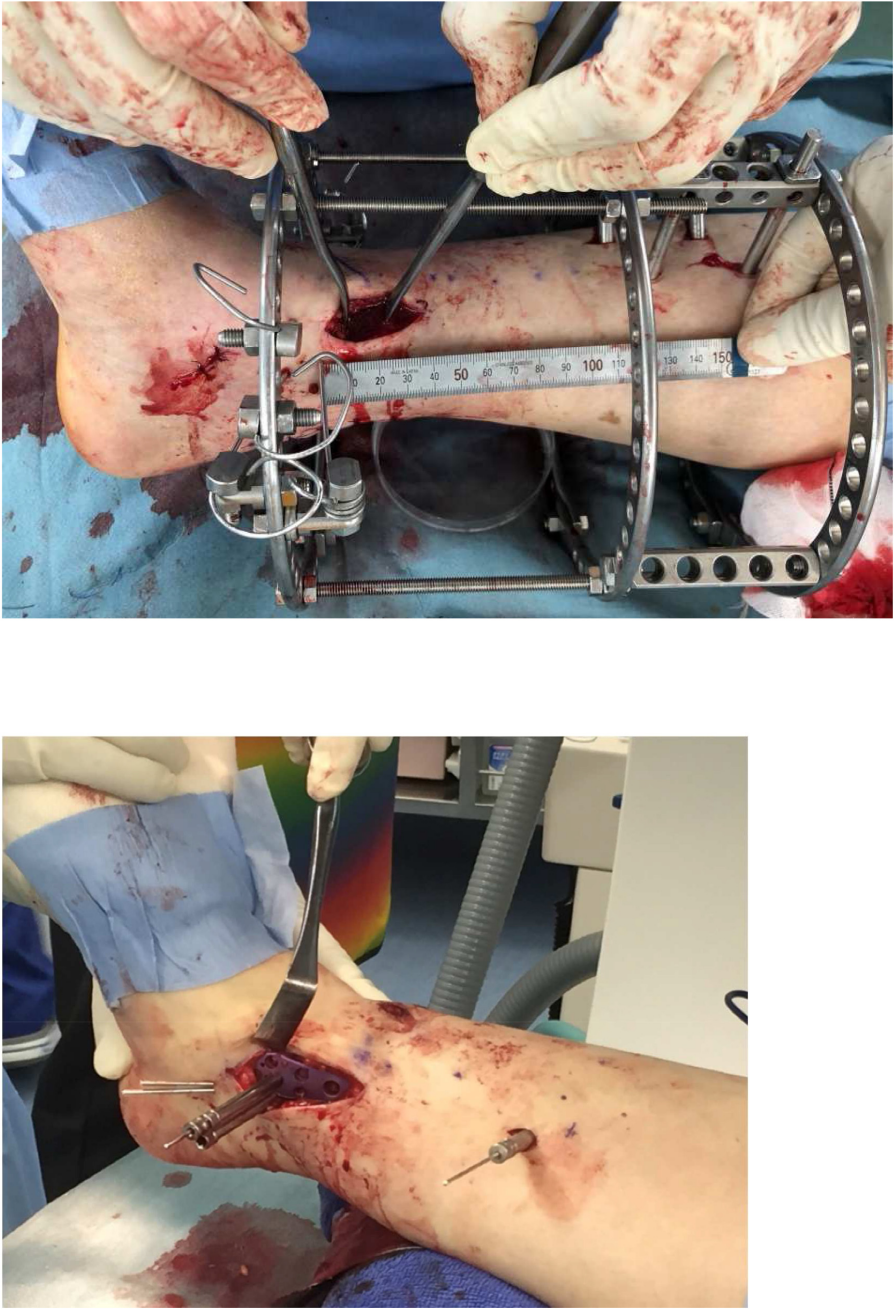
Intra-operative images of surgical approach. **a**. When using a circular external fixator. **b**. A little longer incision is required when using a locking compression plate, and stab incisions are useful for proximal side.

A tourniquet was applied to the ipsilateral thigh, if necessary. The osteotomy line was designed starting from approximately 5 cm proximal to the distal tibial medial malleolus and extending to the center of the distal tibiofibular joint. A skin incision was applied approximately 3 cm long on the medial aspect of the distal lower leg immediately above the starting point of the osteotomy line (Fig. 4 a). In the case of fixed using a locking compression plate, about 5 cm longer incision is needed (Fig. 4 b).

A 1.8- to 2.0-mm Kirschner wire was inserted and used as a guide, the periosteum was detached at minimum along the osteotomy line, and then the osteotomy was carried out with a thin flat-blade bone chisel under fluoroscopic guidance.

#### 2.3.3 Spread the osteotomy gap

Before inserting a laminar spreader, the osteotomy gap was widened with the bone chisel or manipulative procedure to loosen the tension of the surrounding soft tissue. A laminar spreader was inserted into the osteotomy gap, and the osteotomy gap was spread using fluoroscopic imaging.

The osteotomy gap was spread until the lateral articular surface of the talus comes into contact with the medial articular surface of the fibula. When it is difficult to correct the talus position sufficiently, additional surgical technique is required in some cases.

##### 2.3.3.1 Remove bony spurs

Bony spurs may obstruct the valgus and external rotation of the talus. In many cases, bony spurs at the anterior part of the fibula, those at the anterolateral part of the tibia, and those at the lateral part of the talus obstruct talus movement. In the case of these situations after tibial osteotomy, removing the bony spurs is needed through another 3–5 cm incision at the anterior side of the distal tibio-fibular joint toward lateral joint space. We recommend an open approach for the clearance of lateral side as fluid can leak through the osteotomy during arthroscopy.

##### 2.3.3.2 Apply lengthening of the achilles tendon

Spreading the osteotomy gap is the same as acute bone elongation, leading to an increase in soft tissue tension. In particular, lengthening of the Achilles tendon is required after osteotomy in the following cases: varus inclination of the talus is not corrected, anterior subluxation of the talus still remains, and the dorsiflexion angle of the ankle is less than 0° in the knee extension position. We prefer open Z-lengthening of achilles tendon through approximately 5 cm incision at the posteromedial aspect of the achilles tendon than percutaneous triple cut at the view of certainty.

##### 2.3.3.3 Fibular osteotomy

When the talus inclination persists after performing above techniques, fibular osteotomy is taken into consideration. However, in our experience the indication of fibular osteotomy is limited in the cases of traumatic ankle osteoarthritis.

#### 2.3.4 Fix the osteotomized bone with a circular external fixator or a locking plate

After determining the required correction position, the osteotomized bone was fixed with a circular external fixator or a locking plate. Soft tissues surrounding the ankle are thin and tight, with a poor blood supply. Considering that the surgical procedure takes place in such an anatomically unfavorable site and the surgical procedure includes spreading of the osteotomy gap, extremely close attention is necessary throughout the procedure to prevent infection. It is clear that using a circular external frame is minimally invasive procedure for soft tissues, therefore the foot surgery group of our department utilizes a circular external fixator as the first choice for fixation device instead of forcefully fixing the osteotomized bone with internal fixation devices. However, a few patients or surgeons tend to prefer internal fixation devices. One criterion of using a locking plate is “osteotomy gap <15 mm”, but this is not the absolute index and removing the medial tibial cortex bone and cancellous bone is mandatory to allow the plate to be fixed and wound closure achieve without skin tension.

#### 2.3.5 Autogenous bone graft

An iliac crest bone block or cancellous bone was grafted into the osteotomy gap.

#### 2.3.6 Close the wound

A drain was placed at the osteotomy site, and the wound was closed.

### 2.4 Postoperative therapy

The patients were allowed to place the foot of their affected limb on the floor 2 weeks after surgery. Partial weight-bearing was allowed at 6–8 weeks after the surgery. The load level was increased stepwise every week while scheduling the start of full weight-bearing approximately 3 months after the surgery. In general, only active range of motion (ROM) exercises were allowed. Forcefully attempting passive ROM exercises should be prohibited to avoid infection. The external fixator was removed when bone union is achieved (approximately 3–4 months after the surgery).

### 2.5 Statistical analysis

All statistical analyses were performed with EZR (Saitama Medical Center, Jichi Medical University, Saitama, Japan),^14^ which is a graphical user interface for R (The R Foundation for Statistical Computing, Vienna, Austria). More precisely, it is a modified version of R commander designed to add statistical functions frequently used in biostatistics. Continuous variables including the JSSF scale, TAS, MMA, TLS, TTA, and TTS are presented as the mean ± standard deviation and analyzed using paired t-tests. Differences were considered statistically significant at p < 0.05.

## 3 Results

### **3.1** Clinical outcome

Bone healing was achieved in all patients. The JSSF scale improved significantly from 39.9 ± 13.8 (12–65) before surgery to 87.2 ± 7.5 (69–100) after surgery in all stages. All patients could walk independently without ankle pain and then returned to their daily life at the final follow-up. Implant removal was performed in all patients, but no major complications occurred until the last follow-up.

### 3.2 Radiological assessment

The preoperative and postoperative measured values for each parameter were as follows in the order given here: TAS, 84.5 ± 5.0° (68°–93°) and 93.2 ± 5.9° (69°–105°); MMA, 46.5 ± 12.2° (24°–75°) and 32.3 ± 8.3° (15°–54°); TLS, 80.9 ± 6.0° (69°–100°) and 87.0 ± 5.4° (70°–97°); TTA, 9.5 ± 8.2° (0°–28°) and 4.0 ± 4.2° (0°–17°); and TTS, 75.9 ± 8.5° (58°–90°) and 89.9 ± 4.8° (77°–102°). Significant changes were observed after surgery for all parameters, including the TAS, MMA, TLS, TTA, and TTS.

The preoperative and postoperative measured values for each parameter in each stage are shown in Table 1.

**Table 1:** Changes of the JSSF scale and each radiological parameter before and after DTOO.

Stage∗ (n)		Before DTOO	After DTOO	P value∗∗
2 (n = 2)	JSSF scale	49.5 ± 2.1 (48–51)	87.0 ± 5.7 (83–91)	–
	TAS	90.0 ± 1.4 (89–91)	99.0 ± 1.4 (98–100)	–
	MMA	33.0 ± 9.9 (26–40)	22.0 ± 2.8 (20–24)	–
	TLS	82.0 ± 5.7 (78–86)	92.0 ± 5.7 (88–96)	–
	TTA	5.5 ± 7.8 (0–11)	5.0 ± 7.1 (0–10)	–
	TTS	83.5 ± 4.9 (80–87)	93.5 ± 3.5 (91–96)	–
3a (n = 9)	JSSF scale	53.2 ± 7.7 (45–65)	90.0 ± 7.5 (80–100)	<0.0001
	TAS	83.0 ± 4.1 (76–88)	94.0 ± 4.2 (87–101)	<0.0001
	MMA	44.4 ± 7.1 (33–55)	31.3 ± 8.0 (19–43)	0.0003
	TLS	79.4 ± 3.9 (74–85)	88.4 ± 4.1 (80–93)	0.0009
	TTA	8.7 ± 3.0 (5–14)	3.4 ± 2.1 (0–6)	0.0003
	TTS	76.1 ± 4.1 (70–82)	90.7 ± 4.9 (84–100)	<0.0001
3b (n = 30)	JSSF scale	39.0 ± 12.4 (12–59)	87.1 ± 7.7 (69–100)	<0.0001
	TAS	84.4 ± 4.9 (68–91)	92.6 ± 7.0 (69–105)	<0.0001
	MMA	49.7 ± 12.8 (27–75)	33.9 ± 9.2 (15–54)	<0.0001
	TLS	81.2 ± 6.2 (68–100)	86.4 ± 5.7 (70–97)	0.0001
	TTA	13.1 ± 8.3 (0–31)	5.4 ± 4.5 (0–17)	<0.0001
	TTS	72.4 ± 8.3 (58–84)	88.6 ± 5.1 (77–102)	<0.0001
4 (n = 11)	JSSF scale	29.7 ± 13.7 (12–47)	85.0 ± 7.6 (73–93)	<0.0001
	TAS	84.9 ± 6.1 (71–93)	92.9 ± 3.6 (89–100)	0.0004
	MMA	42.1 ± 11.7 (24–60)	30.8 ± 4.9 (23–42)	0.0018
	TLS	81.3 ± 7.5 (69–93)	86.6 ± 5.2 (76–95)	0.004
	TTA	1.0 ± 2.7 (0–9)	0.5 ± 1.3 (0–4)	0.395
	TTS	84.0 ± 5.6 (71–90)	92.2 ± 2.8 (90–98)	0.0005

JSSF scale: Japanese Society for Surgery of the Foot Ankle/Hindfoot Scale.

TAS: Tibial articular surface angle.

MMA: Medial malleolar angle.

TLS: Tibial lateral surface angle.

TTA: Talar tilt angle.

TTS: Tibiotalar surface angle.

∗Tanaka–Takakura classification.

∗∗Paired *t*-test.

### **3.3** Case presentation

#### 3.3.1 Case 1: 61-year-old female, right AOA stage 3a

DTOO with Z elongation of the Achilles tendon and bony spur of anterior fibula resection was performed. DTOO improved congruency of the ankle joint.

The JSSF scale changed from 64 points preoperatively to 95 points 5 years after DTOO.

**Fig. 5:**
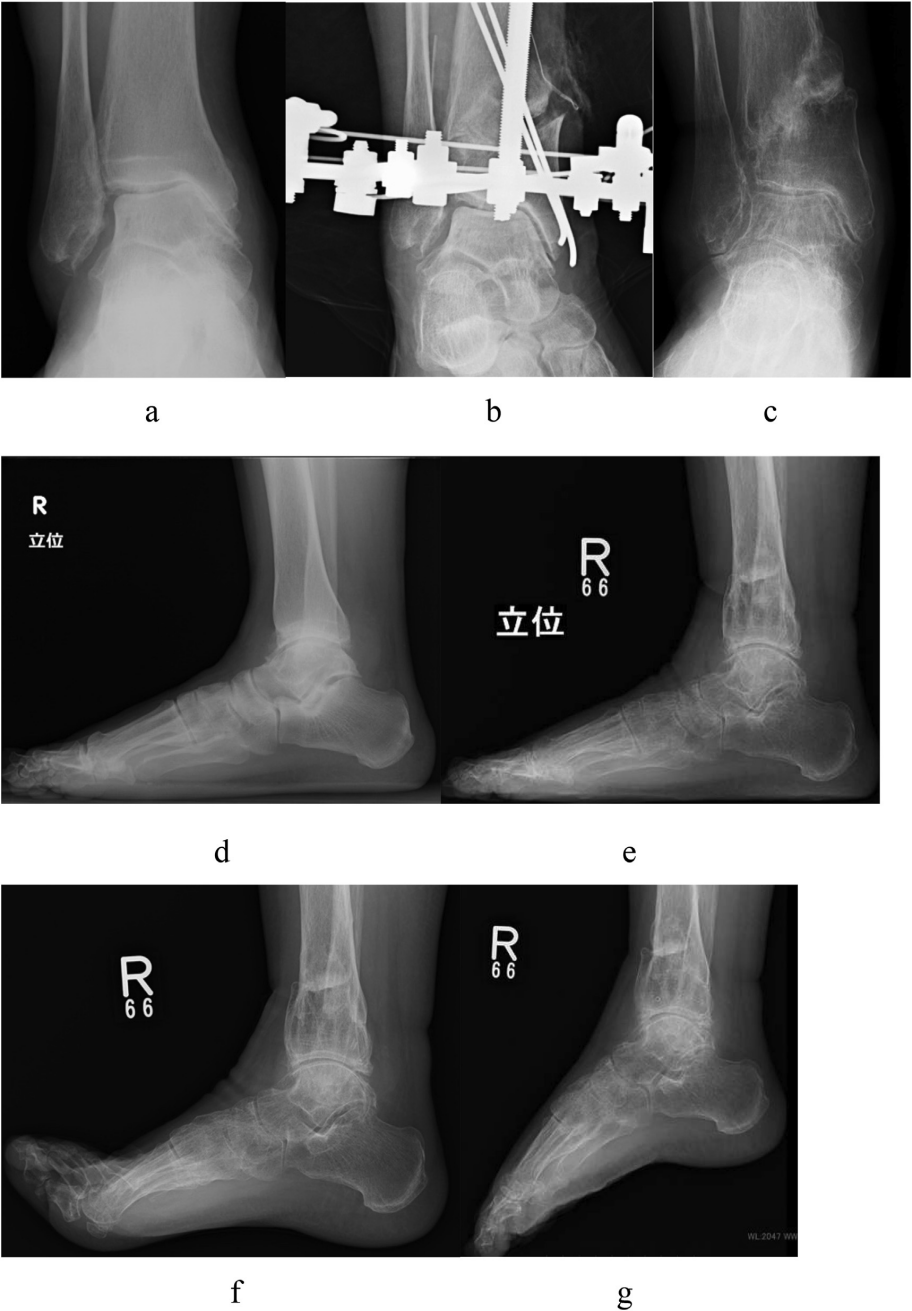
A 61-year-old female. Right ankle osteoarthritis, stage 3a (case 1). This is the typical stage 3a with talar medial shift and a large medial malleolar angle. **a**. Preoperative weight-bearing ankle radiograph (AP view). The medial joint space is narrowing and disappears partly. **b**. Ankle radiograph just after distal tibial oblique osteotomy (DTOO) (AP view). DTOO is performed and fixed using a circular external fixator. **c**. Weight-bearing ankle radiograph (AP view) at the last follow-up. The ankle joint congruency improves after DTOO. **d**. Preoperative weight-bearing ankle radiograph (lateral view). e. Postoperative weight-bearing ankle radiograph (lateral view). The talocrural joint space after DTOO is clearer than before DTOO. **f**. Postoperative ankle radiograph (dorsiflexion lateral view). **g**. Postoperative ankle radiograph (plantar flexion lateral view). The ankle range of motion is maintained after DTOO.

The TAS changed from 84° to 96°, MMA changed from 40° to 23°, TLS changed from 83° to 86°, TTA changed from 8° to 5°, and TTS changed from 76° to 91° after DTOO (Fig. 5).

#### 3.3.2 Case 2: 69-year-old female, right AOA stage 3b

DTOO with Z elongation of the Achilles tendon and bony spur of the anterior fibula and anterolateral tibia resection was performed.

The JSSF scale changed from 52 points preoperatively to 93 points 2 years after DTOO.

**Fig. 6:**
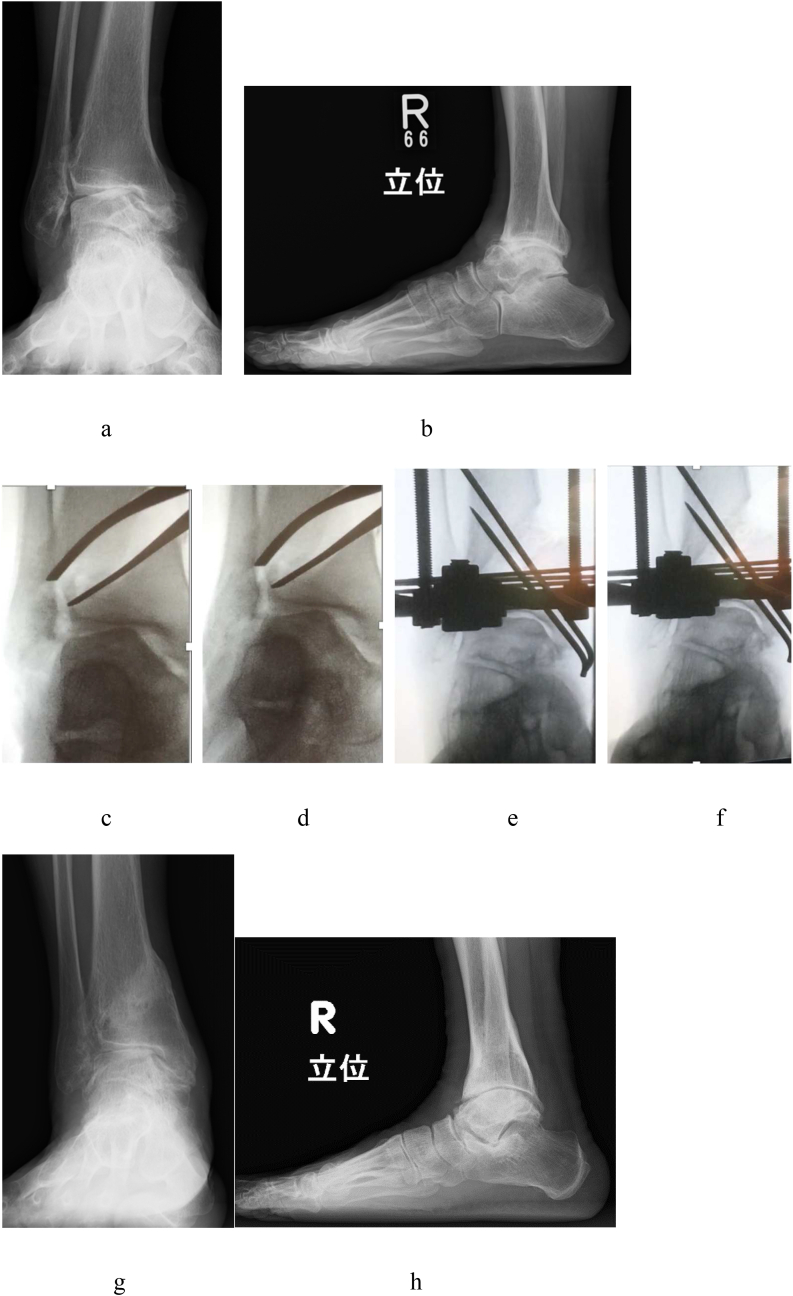
A 69-year-old female. Right ankle osteoarthritis stage 3b (case 2). This is the case of stage 3b without medial talar shift. **a**. Preoperative weight-bearing ankle radiograph (AP view). The medial plafond is caving in due to the large talar tilt. **b**. Preoperative weight-bearing ankle radiograph (lateral view). The talocrural joint seems to be unclear due to the anterior subluxation of the talus. **c**. Intraoperative varus stress fluoroscopy (AP view). **d**. Intraoperative valgus stress fluoroscopy (AP view). Varus–valgus dynamic instability remains after spreading of the osteotomy gap. **e**. Postoperative varus stress fluoroscopy (AP view). **f**. Postoperative valgus stress fluoroscopy (AP view). After lengthening of the Achilles tendon and resection of the bony spurs, dynamic instability seems to disappear. In fact, we change the position of the laminar spreader by trial and error. **g**. Postoperative weight-bearing ankle radiograph (AP view). The talar tilt is corrected, but the incongruity of the tibial plafond and talar dome seems to remain. **h**. Postoperative weight-bearing ankle radiograph (lateral view). The anterior subluxation of the talus is corrected, and the congruity of the talocrural joint seems to improve.

The TAS changed from 83° to 95°, MMA changed from 66° to 36°, TLS changed from 70° to 76°, TTA changed from 21° to 8°, and TTS changed from 63° to 87° after DTOO (Fig. 6).

#### 3.3.3 Case 3: 75-year-old female, right AOA stage 3b

DTOO with Z elongation of the Achilles tendon and bony spur of the anterior fibula, anterolateral tibia, and lateral talus resection was performed.

The JSSF scale changed from 32 points preoperatively to 82 points 2 years after DTOO.

**Fig. 7:**
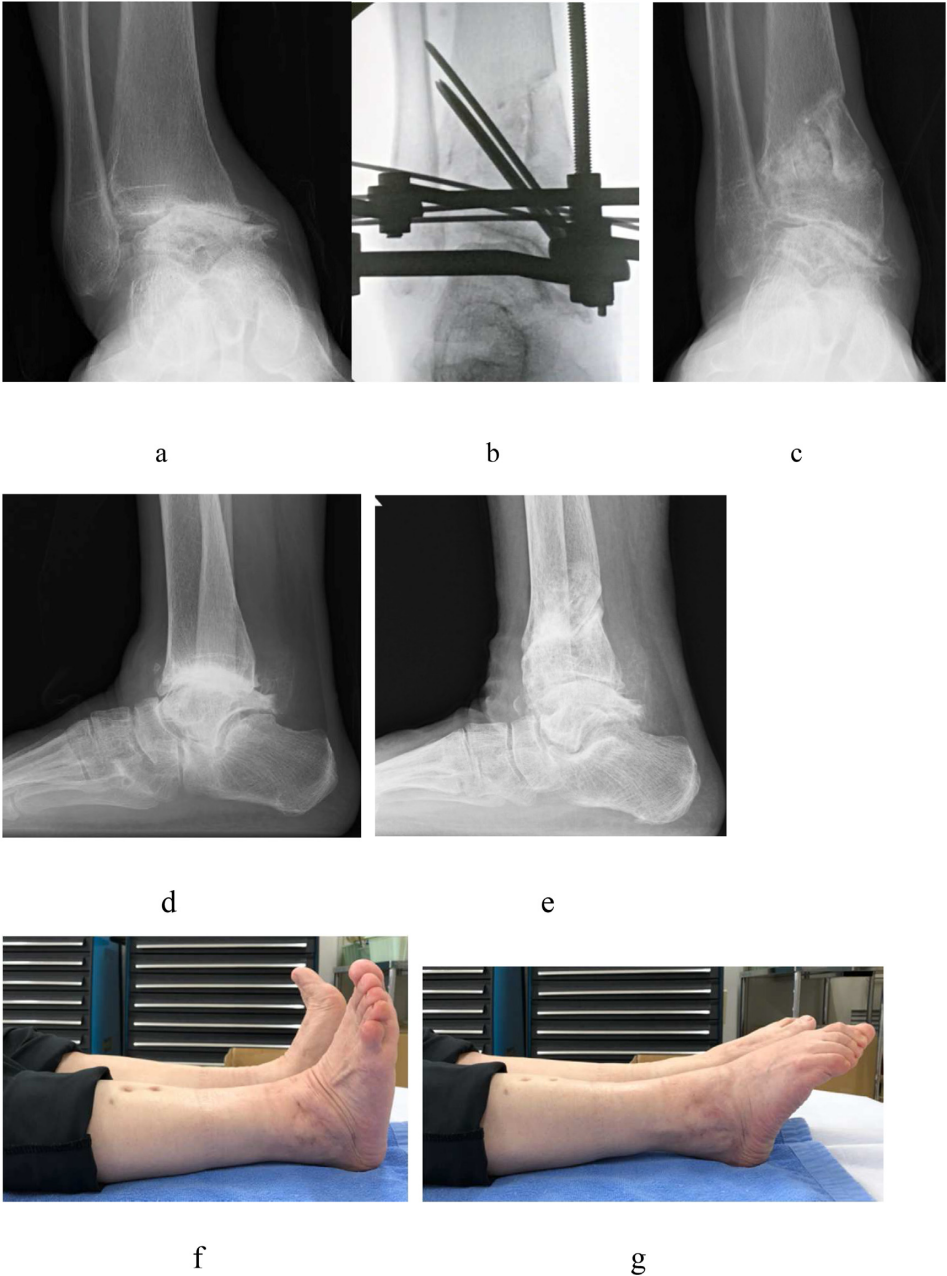
A 75-year-old female. Right ankle osteoarthritis, stage 3b (case 3). Severe varus deformity is observed in this case with an excessive talar tilt. The point different from case 2 is the talar medial shift. Although the talar tilt remains after distal tibial oblique osteotomy (DTOO), the clinical results improve markedly. **a**. Preoperative weight-bearing ankle radiograph (AP view). Severe medial shift and tilt of the talus are observed, and the articular surface of the medial malleoli and medial tibial plafond is caving in. **b**. Intraoperative ankle fluoroscopy (AP view). DTOO was performed with elongation of the Achilles tendon and resection of bony spurs. **c**. Postoperative weight-bearing ankle radiograph (AP view). The medial shift and tilt of the talus were corrected after DTOO, but the incongruity of the tibial plafond and talar dome seemed to remain. **d**. Preoperative weight-bearing ankle radiograph (lateral view). The talus seems to be flattened, and the joint space is unclear. **e**. Postoperative weight-bearing ankle radiograph (lateral view). The joint surface seems to be clear after DTOO. **f**. Postoperative ankle dorsiflexion appearance. Dorsiflexion of the ankle joint in knee extension position is 0°. **g**. Postoperative ankle plantar flexion appearance. Plantar flexion of the ankle joint in knee extension position is 35°.

The TAS changed from 79° to 100°, MMA changed from 75° to 41°, TLS changed from 83° to 87°, TTA changed from 23° to 13°, and TTS changed from 58° to 87° after DTOO (Fig. 7).

#### 3.3.4 Case 4: 65-year-old male, right AOA stage 4

DTOO was performed to maintain ankle ROM and the patient's hope for survival of ankle movement.

The JSSF scale changed from 47 points preoperatively to 87 points 1 year after DTOO.

**Fig. 8:**
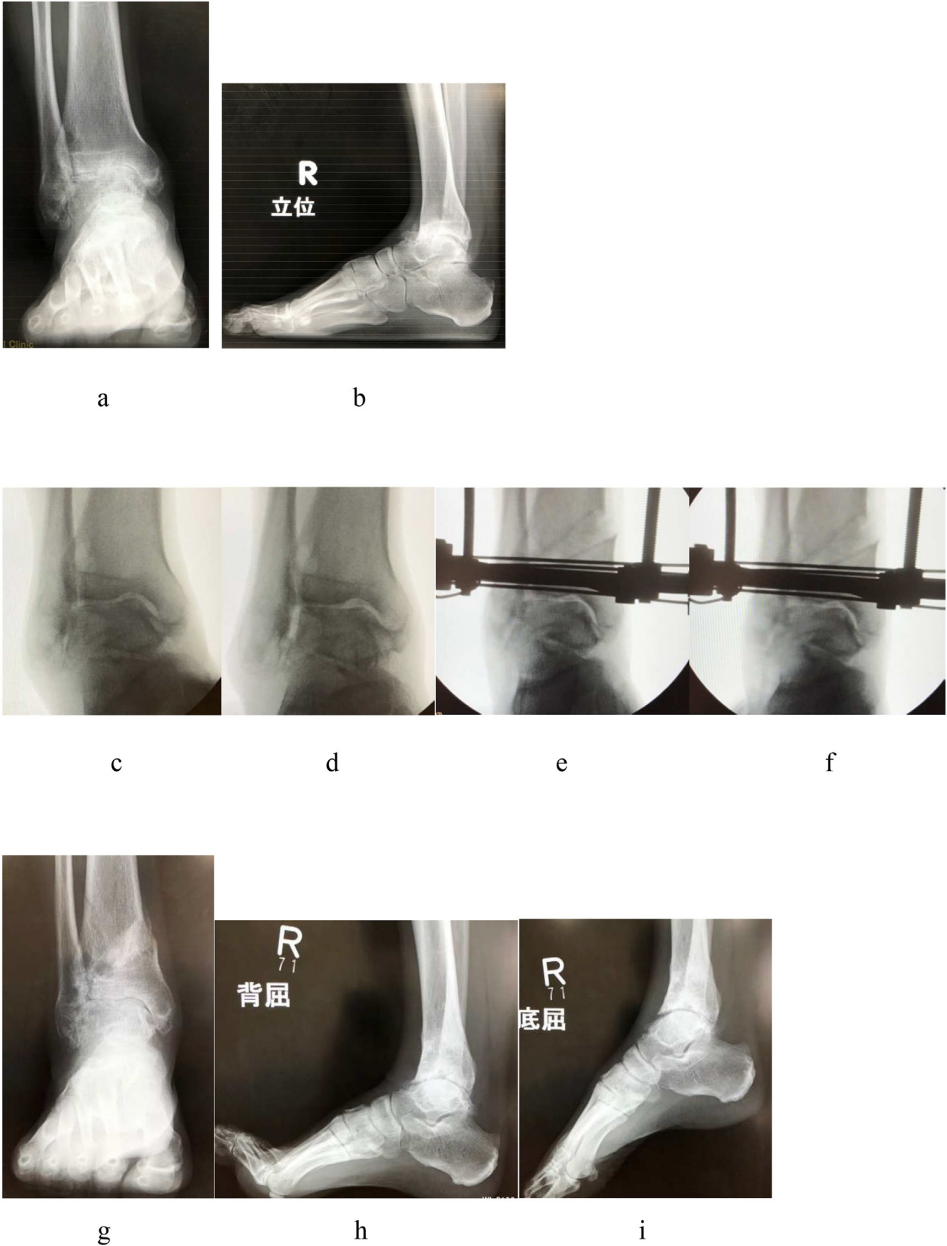
A 65-year-old male. Right ankle osteoarthritis stage 4 (case 4). The preoperative lateral view indicates anterior subluxation of the talus. Distal tibial oblique osteotomy (DTOO) improves the subluxation of the talus and talocrural joint congruency while maintaining the range of motion. **a**. Preoperative weight-bearing ankle radiograph (AP view). The whole joint space seems to be disappeared. **b**. Preoperative weight-bearing ankle radiograph (lateral view). Anterior subluxation of the talus and joint incongruity are noted. **c**. Preoperative varus stress fluoroscopy (AP view). **d**. Preoperative valgus stress fluoroscopy (AP view). Varus–valgus dynamic instability exists. **e**. Postoperative varus stress fluoroscopy (AP view).**f**. Postoperative valgus stress fluoroscopy (AP view). Varus–valgus instability disappears after DTOO. **g**. Postoperative weight-bearing ankle radiograph (AP view). **h**. Postoperative ankle radiograph (lateral view of dorsiflexion). i. Postoperative ankle radiograph (lateral view of plantar flexion). Talocrural joint incongruity improves after DTOO.

The TAS changed from 85° to 89°, MMA changed from 40° to 23°, TLS changed from 81° to 76°, TTA changed from 5° to 2°, and TTS changed from 78° to 90° after DTOO (Fig. 8).

## 4 Discussion

Several literature^15^, ^16^, ^17^, ^18^, ^19^, ^20^, ^21^, ^22^, ^23^, ^24^ on supramalleolar osteotomy (SMO) has been reported in recent years. Some reports have revealed the effectiveness of SMO combined with other additional procedures, such as calcaneal osteotomy, fibular osteotomy, lateral ligament reconstruction, and medial distraction arthroplasty. A large postoperative TTA was reported to be positively correlated with the failure of SMO.^9^^,^^15^, ^16^, ^17^ Otherwise, some authors reported that no radiological outcomes seemed to have a significant influence on clinical results.^25^ Our current study showed the clinical results of each stage according to Tanaka–Takakura classification. To the best of our knowledge, this is the first organized report of DTOO for end-stage AOA. Interestingly, although the JSSF scale was proportionate to the Tanaka–Takakura classification, a large postoperative TTA did not always indicate treatment failure (as in cases 2 and 3). The TTA of stages 3a and 3b significantly decreased after DTOO, but that of stage 4 was too low and had no statistically significant difference before and after DTOO (Table 1).

This study revealed that the TAS, MMA, and TLS significantly changed after DTOO. However, this seems to be natural because DTOO is the tibial opening wedge osteotomy, as most articles about LTO or SMO clarified so far. The different points of our current study were that the TTA of stages 3a and 3b significantly changed after DTOO, and the TTS of stages 3a, 3b, and 4 significantly changed after DTOO. This means that DTOO influenced talar behavior during weight-bearing. One of the correction indices during the DTOO procedure is that the angle between the tibial axis and talar superior surface is approximately 90°. However, we submit that this is not the absolute index and just one of the barometers in light of the essence of DTOO concepts.

We consider that determining the stage based only on the weight-bearing AP view is the controversial point of the Tanaka–Takakura classification. We have experienced a few stage 4 AOAs with a wide range of ankle movements and have performed DTOO for patients with hope of survival of ankle joint movement. Our current study indicates the effectiveness of DTOO for stage 4 AOA (Table 1 and case 4).

DTOO is an advanced osteotomy procedure that has a knack and pitfalls. Complications after DTOO we have come across not in this series were deep infection after internal fixation, prolonged bone union and unexpected arthrodesis.

Finally, DTOO has a clear-cut concept that differs from conventional LTO.^5^, ^6^, ^7^, ^8^ The LTO changes only the alignment, and the shapes of the articular surface do not change. Therefore, ankle joint instability did not improve after LTO. Otherwise, DTOO changes the shape of the articular surface and improves the congruity of the ankle joint. Then, joint stabilization is obtained after DTOO,^15^^,^^26^ and the alignment changes after DTOO, but it is not under control. Because DTOO takes particular note of joint stabilization, not only static radiological evaluation but also dynamic stress tests under fluoroscopy are necessary procedures for accomplishing the concepts of DTOO.

### 4.1 Limitations

The limitations of our current study are as follows: the study was a retrospective study with a small number of patients, different orthopedists handled the surgery, and the results of the radiological assessment were not evaluated for interobserver reliability and intraobserver reliability because radiological assessment was carried out by the same evaluator who was also the corresponding author of this paper and a foot surgery specialist. Moreover, the clinical results shown in the present study are short-term to midterm results. Therefore, our group should continue to observe the clinical course of the subjects in the present study and evaluate the long-term prognosis.

## 5 Conclusion

DTOO may change the articular surface incongruity and dynamic instability of the talocrural joint. Short-term to midterm clinical results from DTOO for AOA are preferable. DTOO is an option applicable to the treatment of patients with AOA.

## Declaration of competing interest

The authors report no conflict of interest with the content of this manuscript.
